# Technological characterization of gold jewellery from the Sogdian tomb of Shi Jun (d. 579 CE) in Xi’an, Shaanxi Province

**DOI:** 10.1038/s41598-020-67788-8

**Published:** 2020-07-01

**Authors:** Panpan Tan, Junchang Yang, Yan Liu, Yaozheng Zheng, Junkai Yang

**Affiliations:** 1 State Key Laboratory of Solidification Processing, Center for Nano Energy Materials, School of Materials Science and Engineering, Northwestern Polytechnical University, Xi’an, 710072 China; 2 Institute of Culture and Heritage, Northwestern Polytechnical University, Xi’an, 710072 China; 3 Xi’an Institute of Conservation and Archaeology on Cultural Heritage, Xi’an, 710068 China

**Keywords:** Solid Earth sciences, Materials science

## Abstract

Northern dynasties (386–581 CE) of China witnessed extensive cultural contacts with the outside world. Several gold objects of this period indicate multiple culture influences. However, very few of them were testified by metallurgic analysis. The gold jewellery including a finger ring and an earring with exotic features were uncovered from the joint elite Sogdian tomb of Shi Jun and his wife of the Northern Zhou dynasty (557–581 CE) in Xi'an. The current study applied multiple non-destructive analyses to investigate the decorative techniques and materials of the two objects. The results showed that both ornaments were made of refined gold. Autogenous welding and brazing were employed for joining the granules of the earring, indicating different technical choices. More interestingly, niello made of silver sulfide was identified as an innovative technology to decorate the finger ring, presenting the earliest evidence of niello inlay in ancient China. It is noteworthy that powders of silver and sulfur were applied separately, deferring from the traditional method of silver sulfide being synthesised prior to being used. These findings help us gain insights into understanding the technical features of early Medieval gold jewellery, as well as the goldsmith’s methods and intentions.

## Introduction

In the period between 386 and 581 CE, the production of gold artefacts in China witnessed a dramatic decline due to a short supply of gold and the significant consumption of gold for religious use^1^. By the time, the country was divided into North China under the control of the Tuoba Xianbei people and South China ruled by Chinese aristocrats. The Northern and Southern dynasties also saw a large influx of foreign immigrants, most of whom were traders from Central Asia and some settled and held official posts. Despite constant political upheavals and warfare, many important changes in material culture took place accompanied with extensive commercial trade and cultural exchange with the outside world. In recent years, about 80 small gold objects were excavated from the elite burials in Shaanxi, Shanxi, Ningxia, Hebei, Henan, and Gansu provinces, as well as Inner Mongolia^2–5^. Drawing from stylistic analysis, some gold artefacts with exotic features such as the granulated ornaments from the Northern Qi (550–577 CE) tomb at Taiyuan and the Northern Wei (386–534 CE) tomb at Datong, Shanxi province were thought to be direct imports and their origins were attributed to Bactria or Sasanian Iran^6–8^. However, none of these assumptions has been testified by metallurgic analysis, not to mention the lack of comparable examples in foreign lands. Only two publications concerned elemental analysis of gold ornaments, for instance, a composition study of a gold finger from the Northern Qi tomb in Taiyuan indicated the use of refined gold^8^; and the XRF results of a granulated earring from another tomb suggested that the autogenous welding was employed for joining the granules^9^. The analyses provided useful data about the metal composition and the soldering technique, but regrettably no detailed discussion was furthered to investigate the technical details and decorative techniques^8,9^. This work arises in response to the need to understand production techniques of early Medieval gold jewellery with debatable origins, and it provides a detailed metallurgic study of technological characterization of two pieces of gold jewellery archaeologically recovered from the joint Sogdian tomb of Shi Jun and his wife in the Chang’an city (modern Xi’an) where diverse influences, cultures and craftsmen interacted between the fourth and the sixth centuries CE. Additionally, we performed a comparative study of the results in space and time, regarding the new evidence of gold jewellery recovered from the Shi Jun tomb in Xi’an, and the existing data of contemporary and subsequent gold work (seventh–eighth centuries CE) in North China and the neighbouring areas.

**Figure 1:**
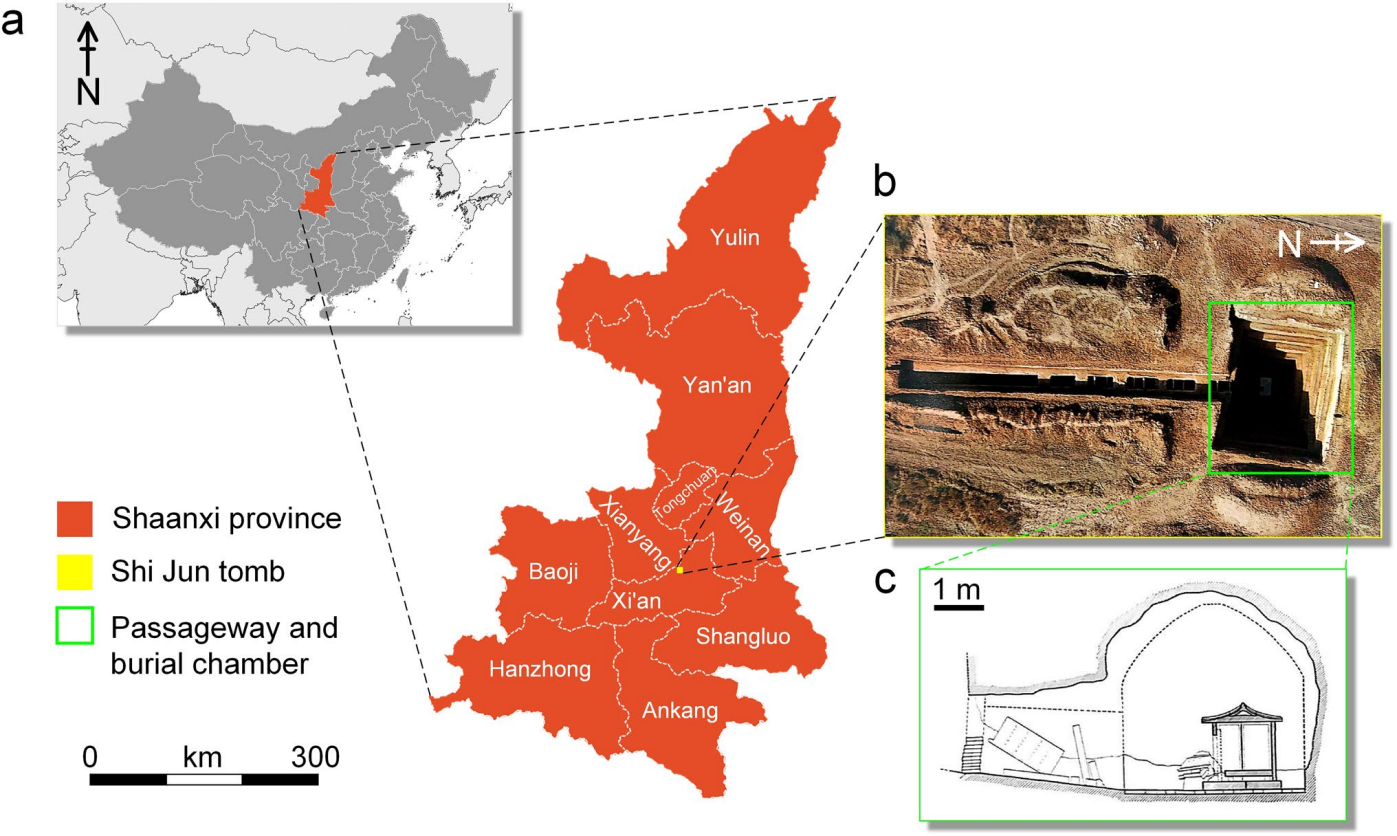
(**a**) Geographical map of Shi Jun tomb. (**b**) Aerial view of Shi Jun tomb^3^. (**c**) Passageway and burial chamber^3^.

**Figure 2:**
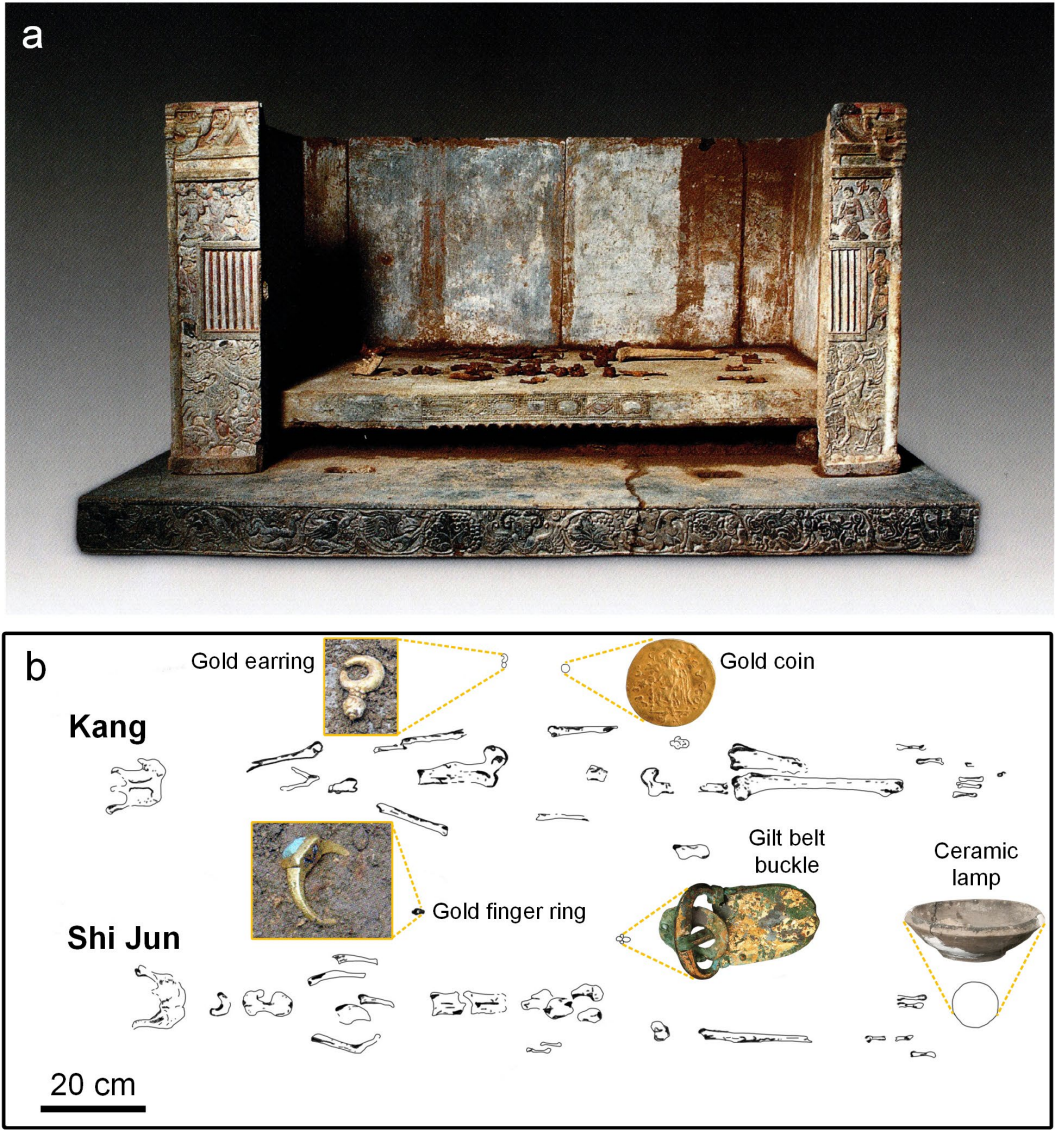
(**a**) The stone funerary couch^3^. (**b**) The excavation positions of the five artefacts on the stone funerary couch (redrawn by Panpan Tan).

The joint tomb of Shi Jun (d. 579 CE) and his wife Kang (d. 579 CE) of the Northern Zhou dynasty (557–581 CE) was excavated in 2003 in the east of Jingshang village, Weiyang district, Xi'an city, Shaanxi province in northwest China (Fig. 1a)^3,10^. The tomb was constructed as a single burial chamber containing a stone sarcophagus in the shape of a Chinese-style house with pictorial reliefs on the outer walls (Fig. 1b,c)^3^. According to the tomb epitaph written in both Chinese and Sogdian^10^, Shi Jun (Sogdian name: Wirkak) was an elite Sogdian born in the ancient Shi State (Kesh, near modern Shahr-i Sabz, south of Samarkand in Uzbekistan), who married Kang (Sogdian name: Wiyusi) from Kang State (modern Samarkand in Uzbekistan) in Senpen in 519 CE. He was appointed Sabao (leader of the foreign communities^11^) of Liangzhou (modern Wuwei in Gansu province), and finally settled in the Chang’an with his family. Regrettably, the tomb was robbed in early time, but five artefacts surprisingly survived, including a gold earring, a gold finger ring, a Byzantine gold coin replica, a gilt belt buckle, and a ceramic lamp^3^ (Fig. 2). The most remarkable objects are the gold earring and the finger ring (Fig. 2b): the former consists of a thick crescent-shaped body and a pearl pendant, between which are two rotatable roundels of tiny granules; the latter contains a rectangle bezel with turquoise inlay, bearing black marks on both sides. The designs of both objects have rarely been seen in the gold ornaments found in other Northern dynasties (386–581 CE) tombs in China. In this paper, the metallurgic analysis of the gold artefacts was undertaken through multiple non-destructive methods, including the structure examination, morphology investigation, elemental analysis, and soldering technique of the earring, and morphology investigation and elemental analysis of both the finger ring and black decorations on it. Whereby these detailed analyses, the production and decorative techniques of the gold jewellery could be well revealed.

### Samples

The original distribution of the two gold artefacts is unclear, as the tomb had been disturbed by robbers. After cleaning, it became possible to see the shape and decorative details of the two gold ornaments.

**Figure 3:**
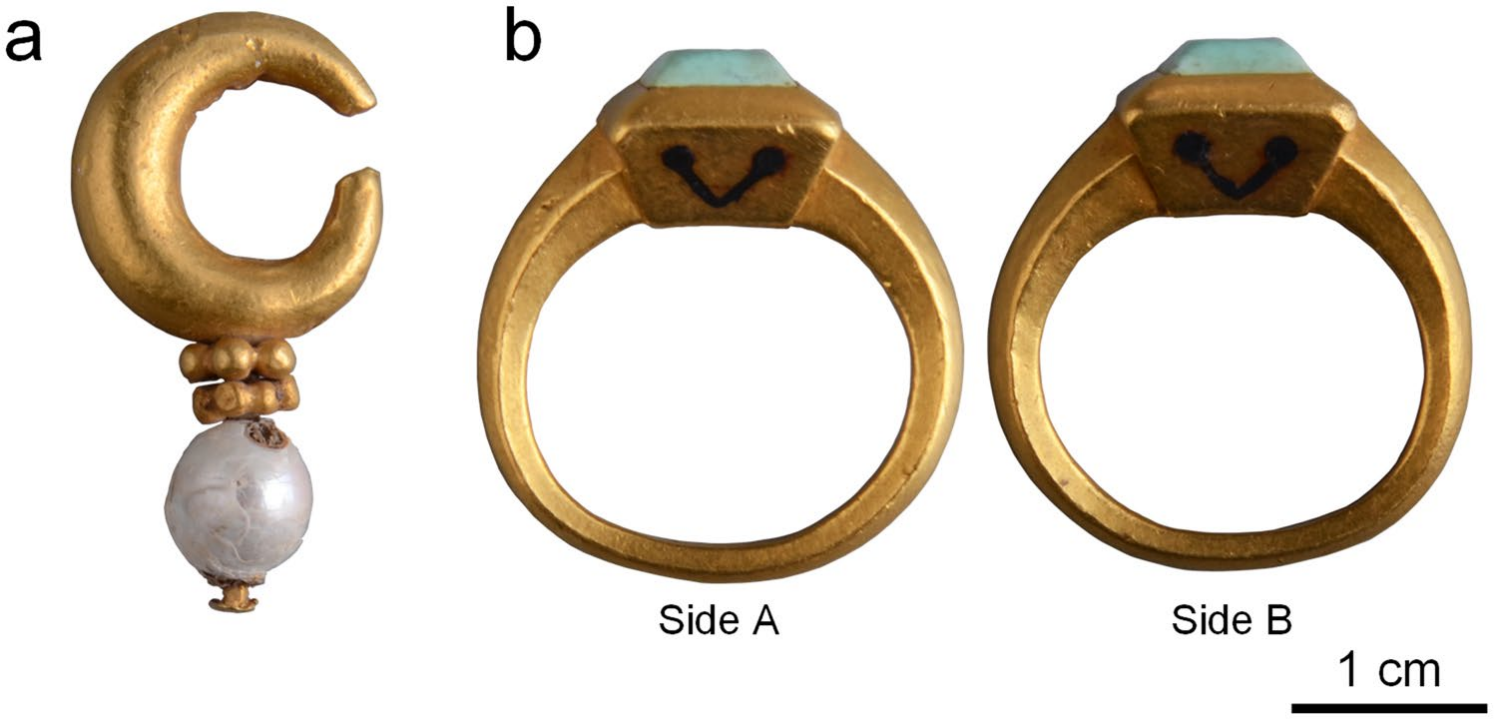
Gold jewellery unearthed from the Shi Jun tomb. (**a**) Gold earring. (**b**) Gold finger ring. Photographer: Chen Wu.

The gold earring (Fig. 3a) comprises three parts: a gold crescent with a thick middle and two thin ends, a gold axle pierced with a pearl, with two small roundels composed of six tiny granules each between the crescent and the pearl, and separated and rotatable granule roundels. The earring measures 2.95 cm in height and 10.95 g in weight.

The gold finger ring (Fig. 3b) is composed of a rectangular bezel with angled sides and a hoop with a well-defined central spine. It is set with a greenish turquoise stone and decorated with V-shaped patterns on each side of the bezel. Both the turquoise inlay and the bezel are well-shaped, with slightly bevelled edges. The finger ring measures 2.42 cm in the outer diameter, 1.91 cm in the inner diameter, and 13.37 g in weight.

## Results and discussion

### The gold earring

**Figure 4:**
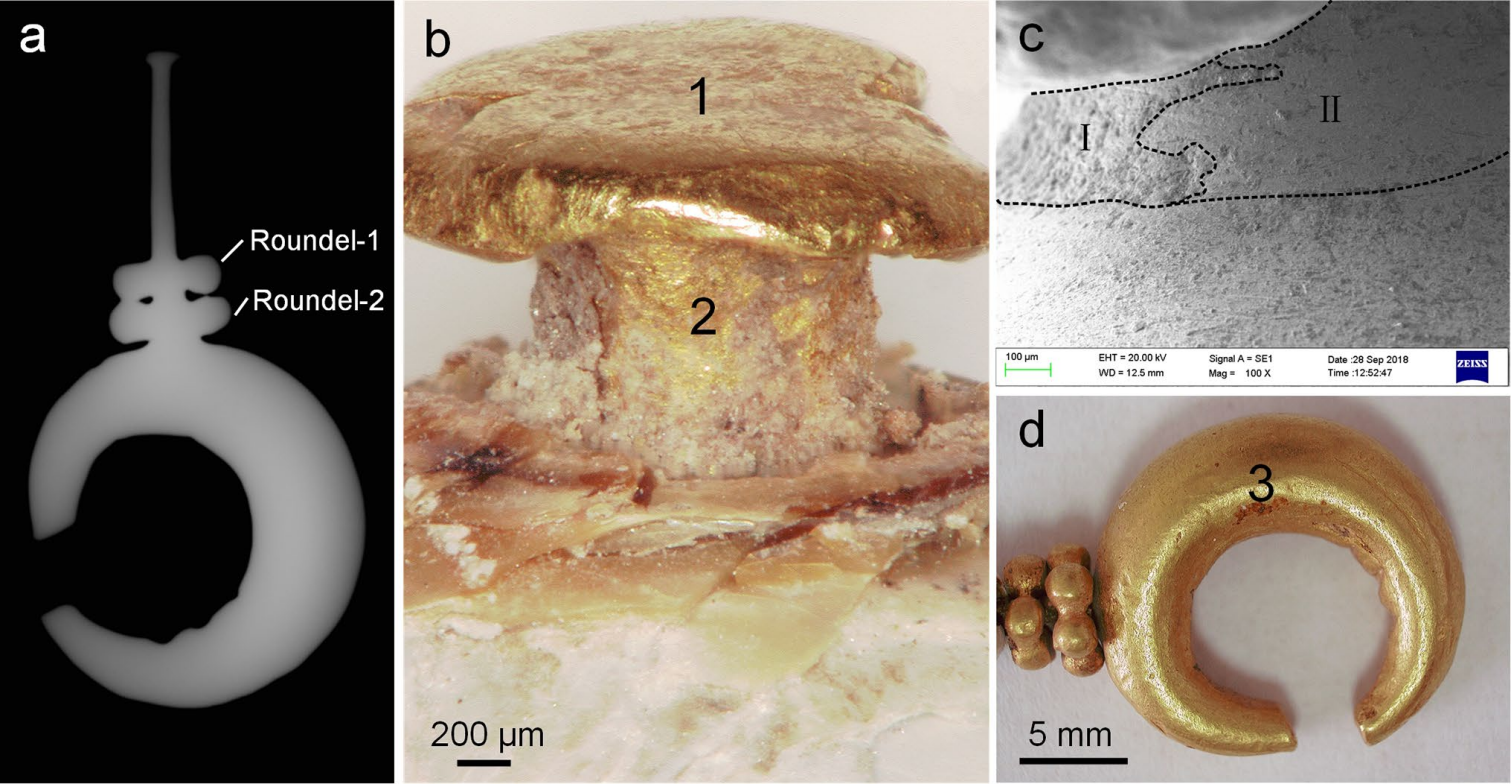
The gold earring. (**a**) X-ray image. (**b**) Micrograph of the axle. (**c**) Connecting part of gold axle and crescent. (**d**) Micrograph of the crescent.

Figure 4a shows the X-ray image of the earring, demonstrating a solid crescent that was probably made by casting; the axle thickens from the end to the joint with the crescent. In the magnified photos, the axle can be seen to have a polygon cross-section, a rough surface, and a hammered end (Fig. 4b), indicating that it had been shaped by hammering. In the area where the axle and the crescent meet, the surface area I is much coarser than area II, reflecting an initial surface in area I and a polished surface in area II (Fig. 4c), which suggests that the axle and the crescent had been bonded by soldering. The elemental analysis further affirms the microscopic observation (Table 1): a very high percentage of gold (Au) and a much lower percentage of silver (Ag) were detected in the axle (Average: Ag:1.8 wt%) and the crescent (Ag:1.6 wt%) (Fig. 4b,d), indicating refined gold was used to produce the gold earring; however, a significantly higher amount of silver (18.4 wt%) and copper (Cu, 4.9 wt%) was found to be present at the joint of the two parts (Fig. 4c), indicating that the axle and crescent were bonded by brazing with the Au–Ag–Cu alloy.

**Table 1:** EDS results for gold earring.

Analysis area	Composition (wt%)			Composition (at%)		
	Au	Ag	Cu	Au	Ag	Cu
Area 1 (n = 3, Fig. 4b)	98.5 ± 0.1	1.5 ± 0.1		97.3 ± 0.1	2.7 ± 0.1	
Area 2 (n = 3, Fig. 4b)	97.9 ± 0.2	2.1 ± 0.2		96.2 ± 0.4	3.8 ± 0.4	
Joint (axle + crescent, n = 3, Fig. 4c)	76.6 ± 6.9	18.4 ± 5.4	4.9 ± 1.5	61.4 ± 9.4	26.5 ± 6.4	12.1 ± 3.1
Area 3 (n = 3, Fig. 4d)	98.4 ± 0.0	1.6 ± 0.1		97.1 ± 0.1	2.9 ± 0.1	
Area 4 (n = 3, Fig. 5a)	98.8 ± 0.1	1.2 ± 0.1		97.8 ± 0.3	2.2 ± 0.3	
Area 5 (n = 3, Fig. 5a)	98.7 ± 0.2	1.3 ± 0.2		97.6 ± 0.3	2.4 ± 0.3	
Joint (4 + 5, n = 3, Fig. 5a)	98.2 ± 0.3	1.8 ± 0.3		96.8 ± 0.5	3.2 ± 0.5	
Area 6 (n = 3, Fig. 5b)	98.8 ± 0.2	1.2 ± 0.2		97.8 ± 0.3	2.2 ± 0.3	
Area 7 (n = 3, Fig. 5b)	97.8 ± 0.4	1.2 ± 0.2	0.9 ± 0.2	95.1 ± 0.8	2.2 ± 0.3	2.7 ± 0.5
Joint (6 + 7, n = 3, Fig. 5b)	96.3 ± 1.9	1.9 ± 1.1	1.7 ± 0.9	91.6 ± 4.1	3.3 ± 1.8	5.1 ± 2.4
Area 8 (n = 3, Fig. 5a)	98.6 ± 0.1	1.4 ± 0.1		97.4 ± 0.3	2.6 ± 0.3	
Area 9 (n = 3, Fig. 5a)	98.3 ± 0.8	1.7 ± 0.8		97.0 ± 1.5	3.0 ± 1.5	
Joint (8 + 9, n = 3, Fig. 5a)	87.0 ± 2.5	8.3 ± 2.8	4.7 ± 0.4	74.5 ± 3.2	13.0 ± 4.2	12.5 ± 1.2
Area 10 (n = 5, Fig. 5b)	86.8 ± 4.0	8.9 ± 3.1	4.3 ± 0.9	74.8 ± 6.3	14.1 ± 4.0	11.2 ± 2.4
Area 11 (n = 3, Fig. 5b)	98.5 ± 0.2	1.5 ± 0.2		97.3 ± 0.3	2.7 ± 0.3	
Joint (10 + 11, n = 6, Fig. 5b)	83.7 ± 4.9	11.2 ± 3.5	5.1 ± 1.8	70.1 ± 7.6	16.8 ± 4.7	13.1 ± 3.9

**Figure 5:**
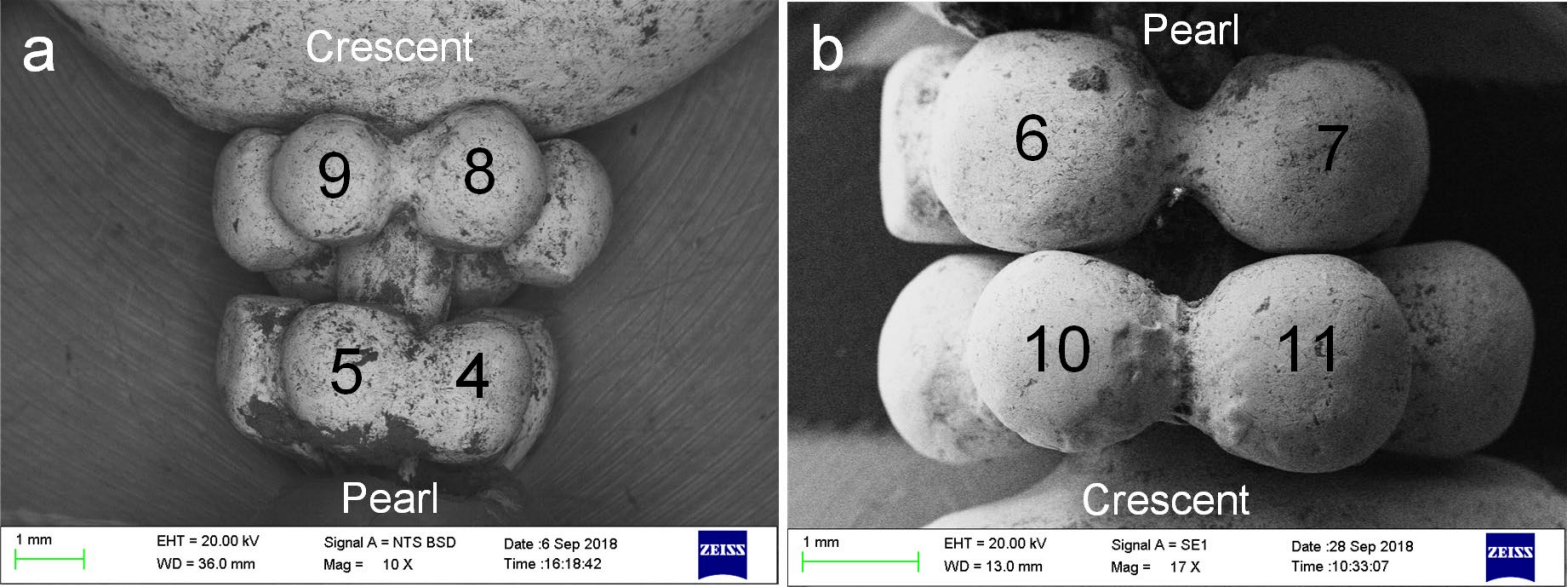
The granule-roundels. (**a**) Gold granule-roundels on one side. (**b**) Gold granule-roundels on other side.

More strikingly, the elemental analysis suggested that different soldering techniques were employed to join the granules of the gold earring. Both the roundels are composed of six small granules, and the average diameter of granules in roundel next to the pearl (roundel–1) (1.98 mm) is very close to that of granules in roundel next to the crescent (roundel–2) (1.96 mm) although roundel–1 is a bit smaller (Fig. 5). In roundel–1, the granules are irregular, much denser with narrow joining areas. Some grains seemed to be fused together (Fig. 5a), probably resulted from overheating during the bonding process. By contrast in roundel–2, the granules are much more spherical and looser with a wider joining area. Filler was clearly observed between the grains (Fig. 5), suggesting that this roundel was made at a relatively lower temperature. The elemental analysis shows that the granules of the two roundels are composed of a similar percentage of Au and Ag to crescent and axle (Table 1) whilst a marked difference is notable at the joints of each roundel. The EDS analysis reflects that the content of Au (96.3–98.2 wt%) and Ag (1.8–1.9 wt%) is very similar to that of the surrounding beads (Au: 97.8–98.8 wt%, Ag: 1.2–1.3 wt%) of roundel–1, indicating that the tiny grains were bonded by autogenous welding^12^ where no solder was used. And interestingly a considerably higher amount of Ag (8.3–11.2 wt%) and Cu (4.7–5.1 wt%) were tested at the joint (Cu and the high content of Ag in area 10 deriving from the solder during the bonding process, Fig. 5b) of roundel 2, suggesting that the granules had been brazed with Au–Ag–Cu alloy (Table 1).

**Figure 6:**
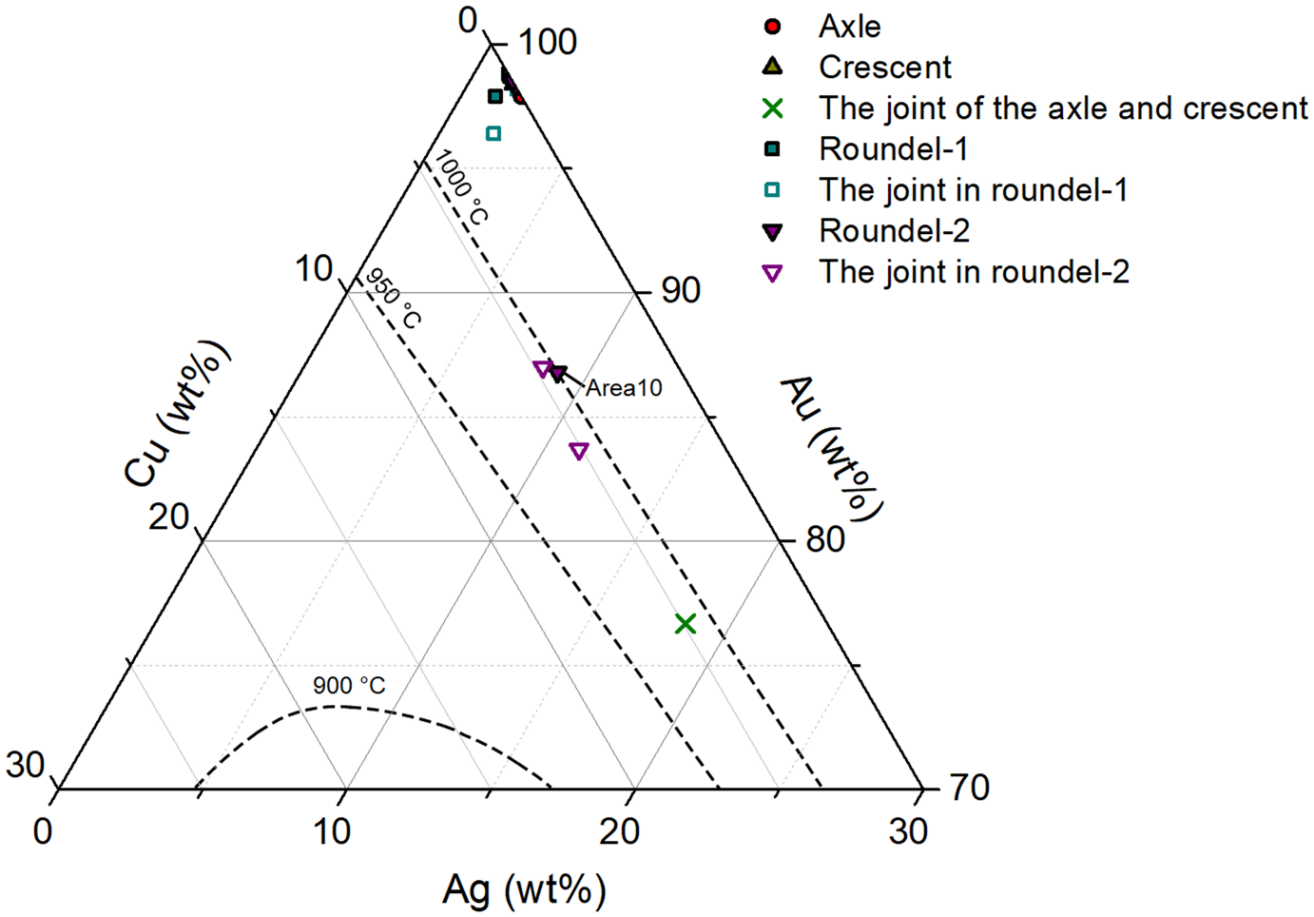
Elemental composition of gold earring. (*liquidus* curves after David A. Scott^13^).

The comparison of compositional data allows us to understand the interrelationship between psychical appearance and technical choice. The co-existence of Au–Ag and Au–Ag–Cu alloys in the joints of gold earring from the Shi Jun tomb indicated that the goldsmiths had different technical choices for soldering, and they might have made a choice with intention. Gold has a melting point of 1,065 °C which could be lowered by adding silver and copper^13^. The addition of silver will improve the hardness and tensile strength of gold^14^. Figure 6 shows the elemental composition of gold earring in the Au–Ag–Cu ternary phase diagram. Solders for roundel–2 and joint between axle and crescent have high amount of silver and copper comparing to the refined gold of other parts of the earring (Table 1), which indicates that silver and copper were added to the gold raw material intentionally to produce a low-melting solder (950–1,000 °C). Moreover, a much higher percentage of silver (18.4 wt%) was detected in the joint of axle–crescent (Table 1), allowing to strengthen the joint and to prevent the roundels and pearl from dropping after long time use.

Clearly, the effects of visual variability in the roundness, dimensions, and joining areas of grains between roundel–1 and roundel–2 were resulted from the use of different soldering techniques. The grains of roundel–1 were joined together by autogenous welding. Autogenous welding is a kind of liquidus phase bonding process, that is, the elements are welded together by heat alone, resulting in no composition change between the joining zone and the metals^12,15^. This technique creates narrow joints^16^ as no solder was used, and an external force was executed to press the grains during the welding process for better bonding. In this way, overheating sometimes happened and the granules lost their roundness. Differently, roundel–2 was made by brazing. The low melting point of Au–Ag–Cu alloy makes the bonding easier, but wider joints were produced^16^.

Why a single piece used two different soldering techniques? It is very likely the two roundels were produced by identical hands, but with different dimensions due to aesthetic preferences. Another possibility is granulation items of this kind were manufactured by two different artisans who employed varied soldering techniques to make the respective parts, and then those pieces were coincidentally assembled on one ornament. In both scenarios the early Medieval goldsmith with exceptional skill in the workshop were able to choose different soldering techniques to fit a specific purpose.

### The gold finger ring

**Figure 7:**
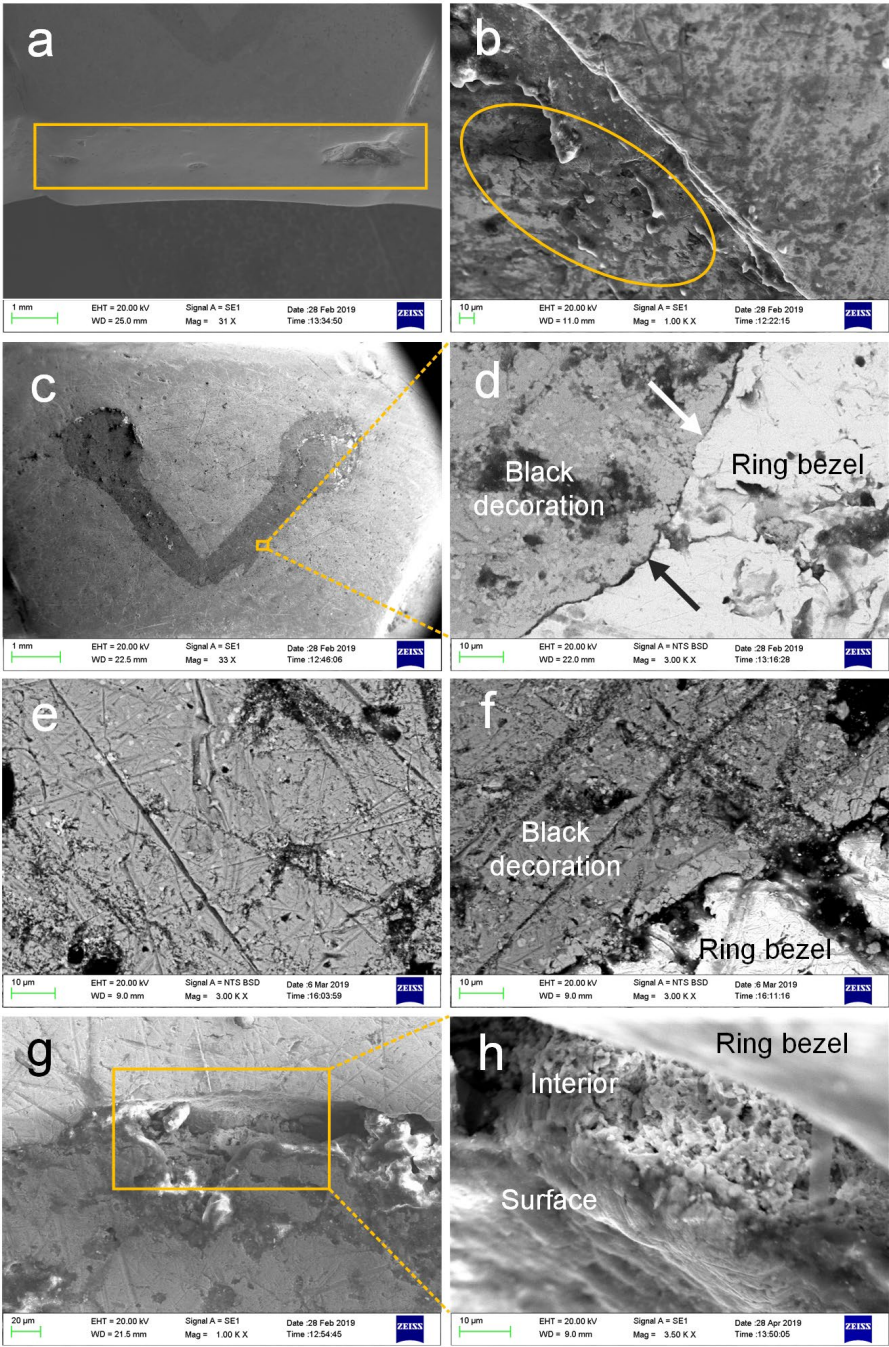
Partially enlarged images of the gold finger ring and SEM images of the black decoration on side A. The casting shrinkage on (**a**) the back of the ring bezel (rectangular frame) and on (**b**) the ring hoop (oval frame). (**c**) the morphology of the black decoration. (**d**) The gap between the black decoration and the ring bezel. (**e**) and (**f**) Bright inclusions in the black decoration (BSE). (**g**) A hole on the edge of the top left of the black decoration. (**h**) The magnified image viewed from the side of the black decoration.

The finger ring was produced by casting, as the defect of shrinkage porosity can be observed at the back of the bezel (Fig. 7a) and on the hoop (Fig. 7b). The EDS results shown in Table 2 indicate that the gold finger ring (Au: 98.6–98.9 wt%, Ag: 1.1–1.4 wt%) has a similar chemical composition to the earring (Table 1), namely, that it was also made of refined gold.

**Table 2:** EDS results of gold finger ring.

Analysis area	Composition (wt%)					Composition (at%)				
	Au	Ag	S	Cl	Br	Au	Ag	S	Cl	Br
Ring bezel (n = 11)	98.6 ± 0.3	1.4 ± 0.3				97.5 ± 0.6	2.5 ± 0.6			
Ring hoop (n = 7)	98.9 ± 0.2	1.1 ± 0.2				97.9 ± 0.3	2.1 ± 0.3			
Black decoration (side A, n = 3)		76.1 ± 1.6	23.9 ± 1.6				48.6 ± 2.1	51.4 ± 2.1		
Inclusions in black decoration (side A, n = 4)	25.7 ± 8.8	57.6 ± 11.0	16.7 ± 2.7			9.5 ± 3.6	50.1 ± 7.0	40.4 ± 3.6		
Interior of black decoration (side A, n = 2)		60.5 ± 5.4	3.0 ± 2.1	4.7 ± 1.9	31.8 ± 1.4		47.6 ± 7.7	7.7 ± 4.9	11.1 ± 4.8	3.6 ± 1.0

**Figure 8:**
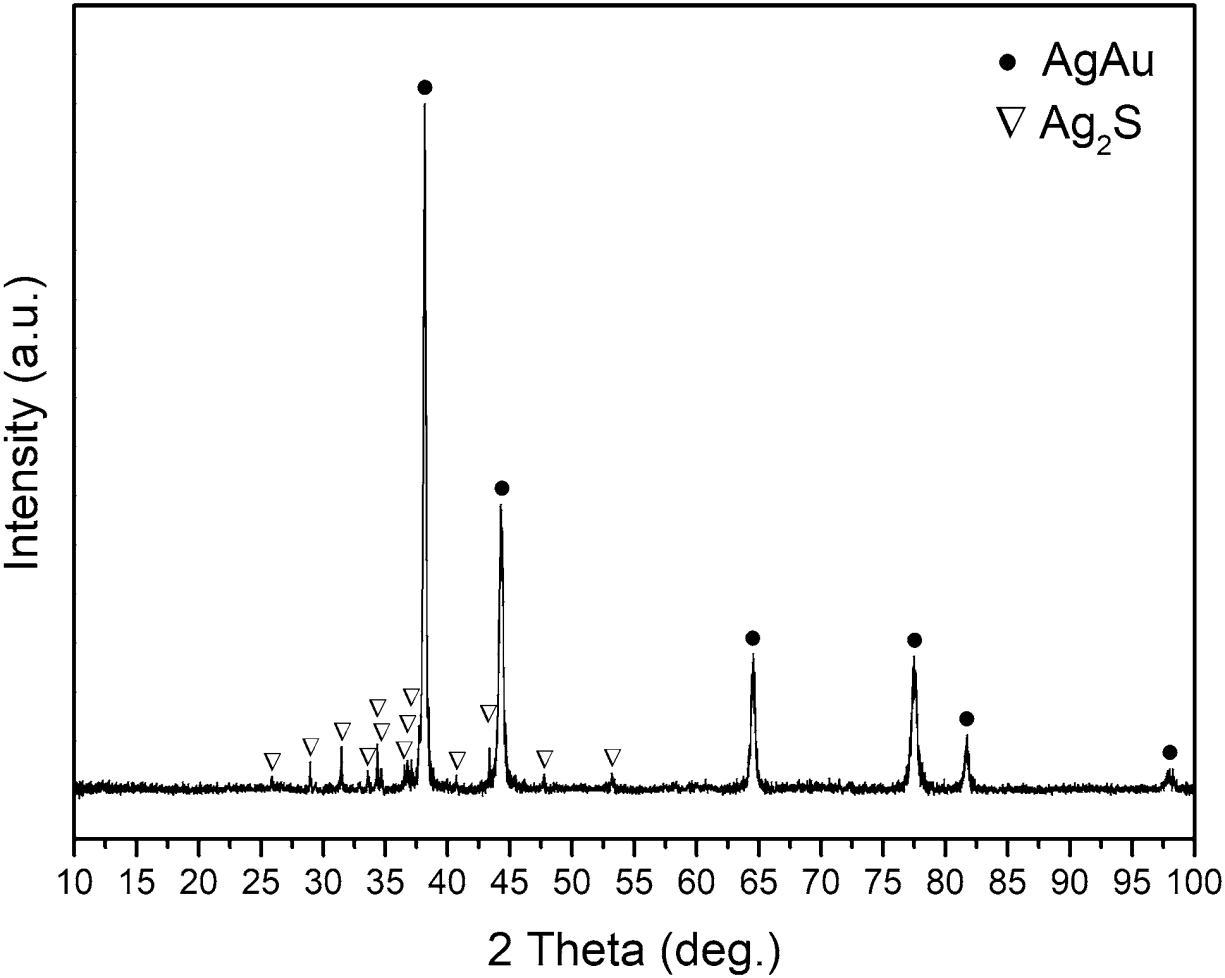
XRD spectrum of the black inlay on side A.

To identify the black marks decorated on the bezel, their morphology, chemical composition and crystal structure were thoroughly analysed. Figure 7c–h show the SEM images of the black decoration on side A. The black pattern can be seen to have a homogeneous and compact morphology (Fig. 7c). On the magnified region in Fig. 7c, a gap can be observed between the black pattern and ring bezel (Fig. 7d), indicating that the black decoration was inlaid in the recess of the bezel. The EDS data shows that the surface of the inlay was chemically homogeneous and made of pure silver and sulfur (S) (Table 2); in addition, the XRD result further confirms that the black mark is monoclinic α-Ag_2_S, acanthite (Fig. 8) (due to the small size of the inlay, the elements of gold and silver in the surroundings were also tested). These results suggest that the Ag_2_S did not originate from the silver corrosion of the product itself, as the corrosion product of archaeological silver is not a single substance (silver chloride is the major one) and black Ag_2_S is primarily formed by long-term exposure to the atmosphere^17^ whilst the marks on the bezel had appeared black when the finger ring was unearthed, as shown in Fig. 2b. The inlay can be observed in detail in the magnified backscattered electron (BSE) images (Fig. 7e,f), with some linear scratches on the surface of the V-shaped decoration attesting to the use of densely inlaid material. Some bright inclusions made of gold (Table 2), differing in shape and size (due to the small size of the inclusions the surroundings were also measured), irregularly distributed in the dark inlay, and even gathering along the edge of the inlay (Fig. 7e,f), indicating that the gold inclusions are probably due to the spread of the gold substrate in the process of polishing. Based on the stated observations and analyses, it can be argued that the compact and chemically homogeneous Ag_2_S was intentionally used to create a contrast with the golden colour of the finger ring, rather than silver corrosion of the product forming post-burial. A similar morphology could be observed on the black decoration of side B, and the same chemical composition was analysed (see Supplementary Information), demonstrating that the black inlays on both sides of the ring bezel were made of the same raw material and produced with a similar technique.

Among inlaying techniques, niello is a black metal sulfide of one or more metals that can be fused or inlaid into the recess engraved in metal including silver, gold and copper-based alloy. Its origin is still obscure^18,19^. The first conclusive evidence of niello dates from the first century CE^18,20,21^. Niello became popular during the Roman Empire, but was uncommon during the tenth–fourteenth centuries CE, following which it came back into favour again in the fifteenth century CE, especially in Italy, finally being superseded by enamel in Western Europe after the fifteenth century CE. Niello survived in some areas of the Middle and Far East^20^. In China, however, neither historical sources nor archaeological items have been found to mention or indicate niello. A silver adornment with the black decoration of the Tang dynasty (608–917 CE) uncovered from the Baotaping cemetery in Chongqing was considered to be niello^22^. However, technically this could not be considered to be true niello since the black sulfide was coated on the metal surface instead of being fixed into the metal recess. Therefore, to date, the niello identified in this paper arguably provides the first evidence of the use of the niello technique in ancient China.

The composition of niello changed over the course of time. During the Roman period, niello was typically composed of the sulfide of one metal, such as silver or copper, for objects made of the same metals^18–20^. There is evidence to suggest that silver–copper sulfide was used intentionally at the end of the fifth century CE, and that silver–copper–lead sulfide was used in Eastern Europe from the eleventh century CE^18,20^. Published niello analyses suggest that silver sulfide was decorated on gold objects between the fourth–fourteenth centuries CE^20,21^. Silver sulfide niello was produced by melting silver and excess sulfur together^19,20,23^. Modern experiments suggest that silver sulfide can only be applied to a metal recess in solid form because silver sulfide is easily decomposed before reaching its melting point (861 °C) in an oxidizing atmosphere; it is thus practical to heat the niello to about 600 °C to soften it, after which it can be applied^20,21,24^. Literary sources from the Early Medieval period described the manufacturing procedure of the niello technique as follows: pulverise the niello, mix it with borax and water, then fill the mixture into the engraved recesses before heating it in situ^19,20^. Strikingly, with regard to the V-shaped black decoration examined in the current study, a composition structure of a grainy outer surface and a porous interior of particle connections could be observed on the side of inlay (Fig. 7g,h). Different from the surface composition (Ag_2_S), elements of silver, sulfur, bromine (Br), and chlorine (Cl) related to the silver corrosion products^17,25^ were measured in the loose structure, besides, more sulfur was detected on the surface (Table 2).

In light of these findings, it can be deduced that the silver sulfide powder could not have been used directly to decorate the gold finger ring. Silver can react with sulfur at room temperature and heating accelerates the reaction; moreover, the reaction could happen at a much lower temperature based on the principle of powder metallurgy. Hence, the niello of the finger ring was probably prepared and applied in situ in the recess on the gold finger ring, as follows: first, silver powder was used to fill in the recess; second, the sulfur powder was spread on the silver powder; then, the inlaying area was slightly heated to accelerate the reaction; finally, the surface of the ring bezel was ground and polished, during which gold particles from the bezel were brought to the surface of the black inlay and scratches left (Fig. 7e,f). During the heating process, sulfur reacted with silver powder to produce a spongy sintered structure of silver sulfide on one hand; on the other hand, the sulfur on the outer surface was oxidised to form sulfur dioxide. In this way, excess sulfur powder was used to guarantee that the silver and sulfur were completely converted to silver sulfide, resulting in a higher percentage of sulfur on the surface (Table 2). Some unreacted silver powder was found in the niello, the bromide and chloride from humus-halide complexes in the soil^25^ entered the interior of the niello to react with the unreacted silver powder, resulting in a porous corroded interior after being buried over 1,000 years.

### Some thoughts about the provenance and origin

From the above elemental analyses, both the gold earring and gold finger ring were made of very good quality of gold. Compared with the very few published data of the Northern dynasties gold, the composition of Shi Jun gold jewellery is distinct from the gold ornaments from the Mausoleum of Emperor Wu (560–578 CE)^26^ in Xianyang, Shaanxi province, and the gold earring from the tomb of Han Zunian (d. 568 CE)^9^ in Taiyuan, Shanxi province. Our result is very similar to the compositions of gold foils from the Mausoleum of Emperor Wu^26^ and the Sogdian tomb of An Jia (d. 579 CE) in Xi’an^27^, as well as the gold finger ring uncovered from the tomb of Xu Xianxiu (d. 571 CE) in Shanxi province^8^. The gold finger ring of Xu Xianxiu was notably bearing the Sasanian or Bactrian glyptics^6^, and therefore was considered as a gift imported by the Sogdian merchant^8^. The data of the Shi Jun gold was different from the neighbouring areas, granulated gold ornaments from the Feng Sufu (d. 415 CE) tomb in Beipiao, Liaoning province^28^ and gold artefacts from Boma cemetery (before the seventh century CE) in Yili, Xinjiang^29^, and it also differed from the later examples, such as the granulated gold ornaments from the coronet of Princess Li Chui in Xi’an (d. 736 CE)^30^.

The archaeological record shows that the gold earrings with crescent body, grain-roundels, and pearl drops were typical Sasanian style. Gold earrings of this type were uncovered in Qasr-i-Abu Nasr (seventh century CE)^31^ and Siraf (c. 650–c. 800 CE)^32^ in Iran. In addition to the Shi Jun finding, similar examples were unearthed from a late fifth century CE tomb in the southern suburbs of the Datong, Shanxi province^33^, and the tomb (seventh–nineth centuries CE) of Tuyuhun (controlled by the Tibetan Tubo Kingdom) in Reshui, Qinghai province^34^. Based upon stylistic analysis, Judith A. Lerner suggested that the gold earring from the Shi Jun tomb probably had a Sassanian origin^6,35^. So far only two objects from the Sackler Museum Collection showed a similar composition with this earring, that is, a gold sword and gold decoration of a gilded buckle dating from the seventh century CE^36^, and both of them belonged to Sasanian Iran.

During the Northern dynasties, new technologies and ideas entered into China with the extensive contacts with Sasanians, Sogdians, Byzantines and other foreign cultures by lands and sea^37,38^. It is not surprising the gold jewelries from the Sogdian tomb in Xi’an showed a range of exotic features. The composition analysis of the Shi Jun earring was pointed to the Sasanian origin, however, the technical details of granulation, especially the soldering techniques, made it difficult to draw a conclusion. Granulation is a historic metalwork originated in West Asia. Extraordinary skill is required for joining the tiny grains. The first evidence of early granulation was recovered in Ur (modern in Iraq), dating back to c. 2,500 BCE^39^. The granulation technique was introduced in northwest China no later than the fourth century BCE, and became very popular in gold work made in the Han period official workshops since the second century BCE^40^. There were three kinds of soldering techniques used in antiquity: (1) autogenous welding; (2) brazing; and (3) copper colloidal fusing. The existing data showed that autogenous welding and brazing were often used to make the granulated gold ornaments found in North China from the fourth century BCE to the tenth century CE while copper colloidal fusing was more common in the gold work found in the Central Asia^41^ and far west to the Mediterranean world^42^.

The results of the Shi Jun earring showed that both brazing with Au–Ag–Cu alloy and autogenous welding were used. The Au–Ag–Cu alloy had a long tradition in ancient China, and it began to be used in the gold granulations in Chinese northern frontiers no later than the fourth century BCE according to the scientific analysis of gold ornaments from the Majiayuan cemetery (fifth–third centuries BCE) in Zhangjiachuan, Gansu province^43^ and Xigou site (third–second centuries BCE) in Hami, Xinjiang^44^. In pre-dynastic China, Chinese workshops were producing gold artefacts and other metal work for the frontier market^45^. The method of autogenous welding was used to make delicate granulated work locally made in the Han period workshops between first century BCE–first century CE^46^. The gold earring from the tomb of Han Zunian in Taiyuan, also used the autogenous welding to bond the granules^9^. Both soldering techniques existed in China for a long time before the Shi Jun finding. Therefore, the earring of Shi Jun was not necessarily imported from Sassanian Iran, it could be a local replica.

So far, more than 10 gold finger rings were found in the elite tombs of Northern dynasties^47^. The ring from the Shi Jun tomb was unique with rectangular bezel and niello inlay, which cannot find a counterpart in contemporary examples. Niello inlay was used as an innovative method for surface decoration of the Shi Jun ring. Unlike paint, the V-shaped marks created by niello inlay could retain for a long time. Niello was extensively used on gold jewellery by Roman goldsmith, and niello of silver sulfide was continuously employed until fifth or sixth century CE^18^. On the Byzantine rings, niello was widely used to enrich the hoop and bezel^48^. However, the niello on the ring of this paper was employed in a very different way from the traditional method, as discussed in the previous section. The compositional data of this finger ring was different from that of Byzantine jewellery between fourth–seventh century CE^49^. Although the question of the origin of the Shi Jun ring admits of no conclusive answer due to insufficient data of comparable examples, it is certain that the niello inlay was purely foreign to ancient Chinese prior to the Northern dynasties, and the scientific data in this research provides informative evidence for future research.

## Conclusion

Early Medieval Age gold jewellery from the Sogdian tomb in Xi’an, northwest China, presents with a variety of manufacturing techniques and innovative methods. The compositional analysis showed both the gold earring and finger ring were made of refined gold. The microanalysis conducted on the gold earring in the current study revealed that different soldering methods were employed for jointing several parts of the earring based on the functions of the components. The Au–Ag–Cu alloy with high silver was used to strengthen the joining area of crescent–axle. No solder alloy was identified in the joining areas of roundel-1, while the joining area of the roundel–2 used Au–Ag–Cu alloy. These two soldering techniques were applied to produce roundels with different dimensions. The microscopic examination and elemental analysis, together with the XRD analysis, demonstrated that the black V-shaped marks on the finger ring bezel could be identified as niello inlay, employing powdered silver and sulfur separately, providing the first evidence of the niello technique ever discovered in ancient China.

It is difficult to provenance the exact place where these two objects were made due to insufficient data of parallels in the Sasanian or Byzantine gold work between the fourth and sixth centuries. Judging from the stylistic and technical features (granulation and niello), both the earring and the finger ring represent a fusion of multiple cultural influences. The crescent earrings with granulated roundels were widespread in northern China between the fifth and nineth centuries CE, and their prototypes could be traced back to the Sasanian Iran. The laboratory analysis in this study thus provides some clues for understanding the technical details of granulated gold in the sixth century CE. The granulated gold from the fourth century BCE in northwest China normally employed Au–Ag–Cu alloy for soldering and more delicate granulated gold of the first century CE in central China used autogenous welding for bonding granules. These two soldering techniques were continually used for the earring found in Shi Jun’s tomb, as affirmed by our elemental analysis. It is very likely that the gold earring was locally made in a foreign style. Another important finding of the current research pertains to the niello inlay, which was found to have been employed for the surface decoration of the gold finger ring in an unconventional way. Further analyses of granulation and niello artefacts, and integrated studies of pearl and turquoise decorated on the gold earring and finger ring studied here, are required in order to clarify the origins of the two pieces of jewellery found in Shi Jun’s tomb.

## Methods

### Microscopic analysis

The samples were photographed using a 3D digital microscope (HIROX KH-7700 3D, Japan) with large scene depth. The observations were carried out with high-resolution HD (1,600 × 1,200), as well as a multi-focus system.

### X-ray radiography

X-ray images were obtained using an X-ray detector (GILARDONI ART—GIL350/6, Italy), and a digital imaging system (DUERR CRNet/HD-CR 35 NDT Plus, Germany) equipped with an imaging plate (HD-IP Plus, Germany) of 10 cm × 14 cm, with a pixel size of 25 μm. Operating conditions for the X-ray detector involved an energy setting of 160 kV, electric current settings of 5 mA, and exposure time settings of 120 s. The working distance was 80 cm.

### Scanning electron microscopy with energy dispersive spectrometer (SEM–EDS)

The morphology analyses were performed by a SEM (ZEISS EVO MA 25, Germany) for the purpose of observing the surface related to the manufacturing process. The composition analyses were undertaken by EDS (Oxford X-max 20, UK). The SEM–EDS operating conditions were in line-avg mode with 20 kV accelerating voltage and an EDS working distance of around 8–9 mm. At least two micro-areas were analysed in every zone, and the elemental results detected were averaged and normalised.

### X-ray diffraction (XRD)

The diffraction patterns of the mineralogical composition were collected by high-resolution XRD (Rigaku SmartLab 9 kW, Japan), which can be used to study solid objects with various sizes without the need for sample preparation. The system was equipped with the D/teX Ultra detector with Cu anode (45 kV, 200 mA), using a continuous scan mode in the range of 2θ from 10° to 100°.

## Supplementary information

Supplementary file1 (PDF 448 kb)

## Data Availability

All data generated or analysed during this study are included in this published article and its Supplementary Information files.
