# Supplementary material for: Technological characterization of gold jewellery from the Sogdian tomb of Shi Jun (d. 579 CE) in Xi’an, Shaanxi Province
Source: Sci Rep. 2020 Jul 1;10:10804. doi: 10.1038/s41598-020-67788-8 (PMC7330028; doi:10.1038/s41598-020-67788-8)
Supplement: Supplementary file 1 — Supplementary file1 (PDF 448 kb) [file 41598_2020_67788_MOESM1_ESM.pdf]

Supplementary Information

Technological characterization of gold jewellery from the Sogdian tomb of Shi Jun (d. 579 CE) in Xi'an, Shaanxi Province

Panpan Tan[1], Junchang Yang[2]*, Yan Liu[2]*, Yaozheng Zheng[1] & Junkai Yang[3]

[1]State Key Laboratory of Solidification Processing, Center for Nano Energy Materials, School of Materials Science and Engineering, Northwestern Polytechnical University, Xi'an, 710072, China.
[2]Institute of Culture and Heritage, Northwestern Polytechnical University, Xi'an, 710072, China.
[3]Xi'an Institute of Conservation and Archaeology on Cultural Heritage, Xi'an, 710068, China.

*Correspondence to: yangjunchang@nwpu.edu.cn and yliu2018@nwpu.edu.cn

This file includes:

Scientific analysis of black decoration on side B of gold finger ring from the tomb of Shi Jun
Figure S1 to S2
Table S1

Scientific analysis of black decoration on side B of gold finger ring from the tomb of Shi Jun

Fig. S1 shows the SEM images of the black decoration on side B. The surface of the black decoration is dense and scratches and some damage presence on it (Fig. S1a). The outline of the decoration is more distinctive in the backscattered electron image, the boundaries between the black decoration and gold bezel, scratches and damage are covered by dark pollutants (Fig. S1b), indicating the black “V”-shaped decoration was probably inlaid in the recess engraved on the gold bezel. The composition analytical result shows that pure silver and sulfur present in the dense surface (Table S1); only one monoclinic α-Ag_2S, acanthite was tested by the XRD (due to the small size of the inlay, the elements of gold and silver in the surroundings were also tested) (Fig. S2). In the magnified backscattered electron images (Fig. S1c and d), with some linear scratches on the surface of the V-shaped decoration attesting to the use of densely inlaid material. Some bright inclusions made of gold (Table S1), differing in shape and size (due to the small size of the inclusions the surroundings were also measured), irregularly distributed in the dark inlay, and even gathering along the edge of the inlay (Fig. S1c and d). Viewed from the side, a composition structure of a grainy outer surface and a porous interior of particle connections could be observed on the side of inlay (Fig. S1e and f). In the porous interior, elements of silver, sulfur, chlorine, and bromine were analysed (Table S1).

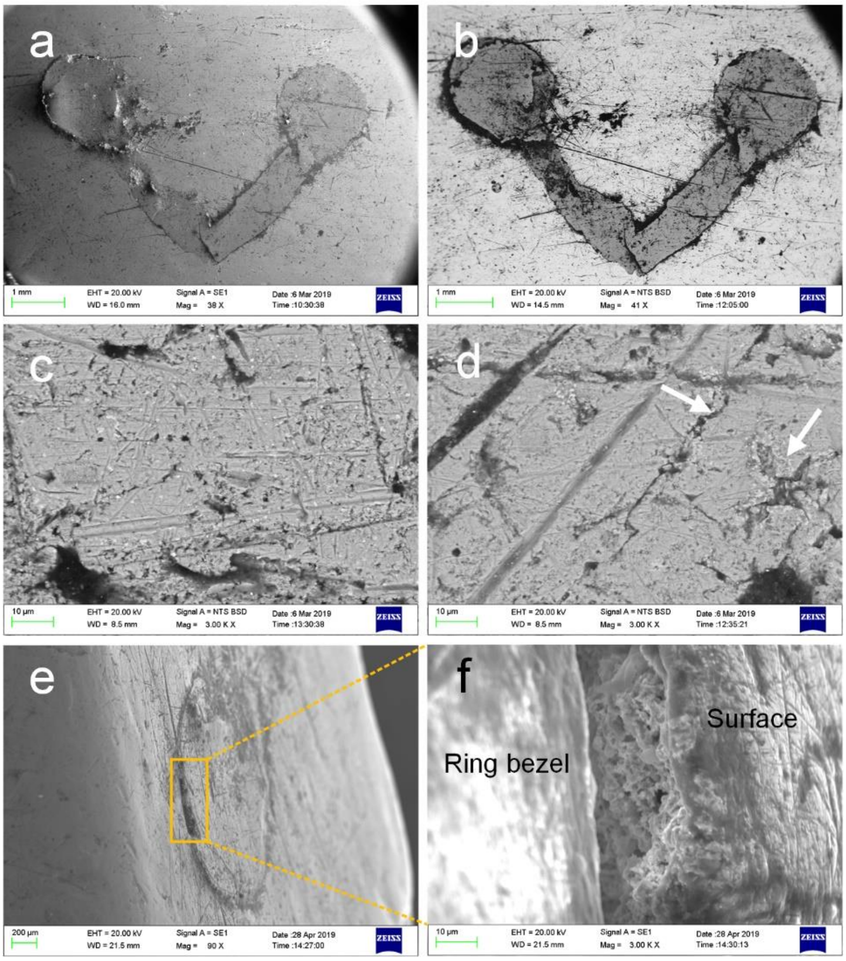

Figure S1. The SEM images of the black decoration on side B. (**a**) the secondary electron image of the black decoration. **(b)** the backscattered electron image of the black decoration. **(c)** and **(d)** bright inclusions in the black decoration. **(e)** a hole at the edge of the top right of the black decoration. **(f)** the magnified image viewed from the side of the black decoration.

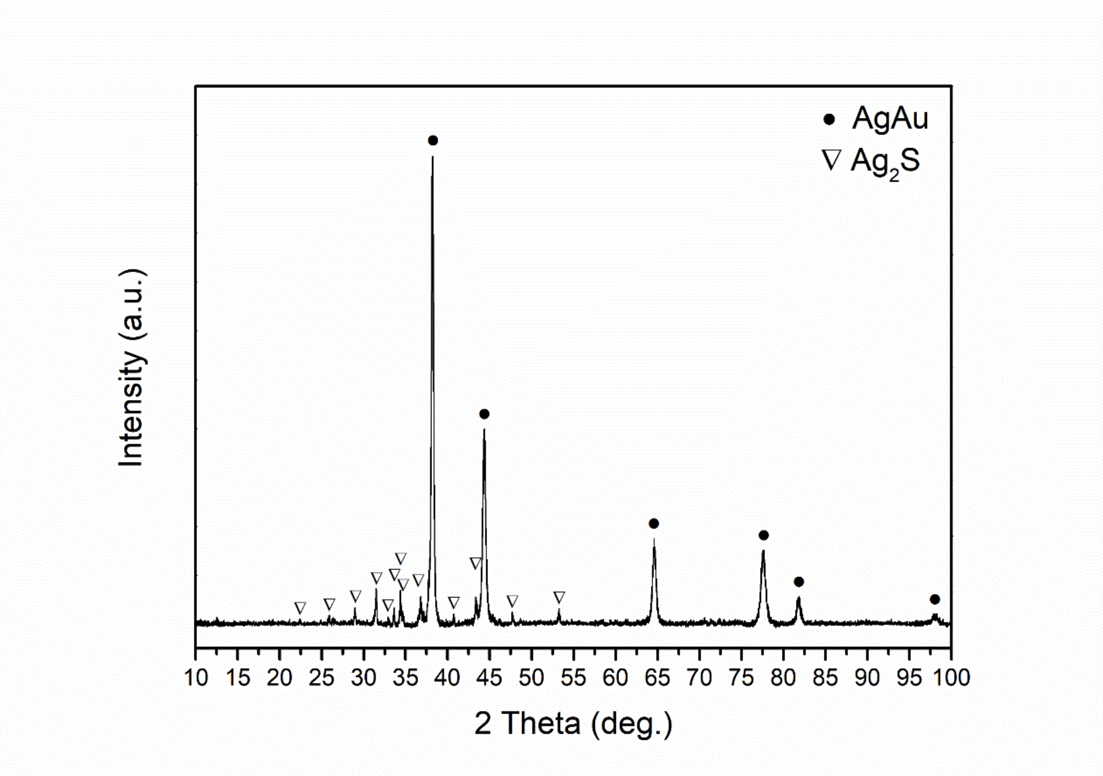

Figure S2. XRD spectrum of the black inlay on side B.

Analysis area	Composition (wt%)					Composition (at%)				
	Au	Ag	S	Cl	Br	Au	Ag	S	Cl	Br
Black decoration (side B, n=4)		75.5±1.2	24.5±1.2				47.8±1.6	52.5±1.6		
Inclusions in black decoration (side B, n=5)	23.2±8.0	60.2±8.0	16.6±3.1			10.0±4.1	46.9±6.4	43.1±5.4		
Interior of black decoration (side B, n=3)		73.3±6.0	6.0±4.9	6.1±2.2	14.6±11.1		55.7±2.8	14.8±11.8	14.0±4.6	15.5±12.6

Table S1. EDS results of black decoration on side B.
